# Differential motility parameters and identification of proteomic profiles of human sperm cryopreserved with cryostraw and cryovial

**DOI:** 10.1186/s12014-019-9244-2

**Published:** 2019-06-19

**Authors:** Shanshan Li, Lei Ao, Yaping Yan, Jiang Jiang, Bingbing Chen, Yanchao Duan, Fei Shen, Jinbao Chen, Briauna Inglis, Renmin Ni, Weizhi Ji, Wei Si

**Affiliations:** 1 Yunnan Key Laboratory of Primate Biomedical Research, Institute of Primate Translational Medicine, Kunming University of Science and Technology, Kunming, Yunnan China; 2 Kunming Sino-UK Angel Women’s & Children’s Hospital, Kunming, Yunnan China; 3 Department of Obstetrics, The First People’s Hospital of Yunnan Province, Kunming, Yunnan China

**Keywords:** Human sperm, Proteomic profile, iTRAQ, Cryopreservation, Cryostraw, Cryovial

## Abstract

**Background:**

Although sperm cryopreservation has been widely used in human reproductive medicine as an integral infertility management in infertility clinics and for banking sperm in sperm banks, the freezing/thawing protocols are not optimal. The freezing and thawing processes result in changes at both structural and molecular levels, some even detrimental, in human sperm when compared with fresh sperm. The change of sperm proteins after cryopreservation may play negative roles for fertilization and early embryo development. Conventionally, cryostraws (CS) and cryovials (CV) are the most widely used cryopreservation carriers (CPCs) for human sperm cryopreservation accompanied with the use of egg yolk free commercial media. However, the influence of cryopreservation on the proteomic profile of human sperm preserved with the two CPCs is unknown. Therefore the purpose of the present study was to compare the frozen-thawed motility, investigate the proteomic profile of human sperm cryopreserved with the two types of CPCs, and identify the susceptible proteins that play key roles for sperm function and fertility.

**Methods:**

The present study compared the cryosurvival of human sperm frozen with the two different CPCs and identified the sperm proteomic changes by using the isobaric tags for relative and absolute quantification labeling technique coupled with 2D LC–MS/MS analysis after freezing and thawing.

**Results:**

Our results indicated that sperm cryopreserved with CV showed higher values for percentage of motile sperm and forward activity rate than those with CS. Compared to fresh sperm, 434 and 432 proteins were differentially identified in human sperm cryopreserved with CS and CV, respectively.

**Conclusion:**

The proteomic profiles of human sperm are greatly affected by cryopreservation with either type of CPC. GO analysis revealed that most of the differentially identified sperm proteins enriched in the extracellular membrane-bounded organelles, cytoplasm and cytosol. In addition, 106 susceptible proteins having known identities related to sperm functions were identified. In general, cryovial seems to be the preferred CPC for human sperm cryopreservation based on the post-thaw motility parameters and the effect on sperm proteomic profiles. These results are beneficial for the insight into the understanding of the cryoinjury mechanism of sperm and the development of human sperm cryopreservation strategies.

**Electronic supplementary material:**

The online version of this article (10.1186/s12014-019-9244-2) contains supplementary material, which is available to authorized users.

## Background

Human sperm cryopreservation is an important technique for infertility management in human reproductive medicine and for male fertility preservation in cases of malignancy treatments [1, 2]. However, current sperm freezing protocols are not optimal and the freezing and thawing process results in inevitable changes, both at structural and molecular levels, most of them are detrimental to sperm and therefore, cryopreserved sperm have shown decreased frozen-thawed motility, cell viability and fertilizing ability compared to fresh sperm [3]. It has been proven that physical and chemical factors including rapid change in temperature, intracellular ice formation, osmolality increase, oxidative stress, pH change, and adenosine triphosphate (ATP) production disturbance are the main causes of sperm cryoinjuries during the freezing and thawing process [4–12]. However, the mechanism of sperm cryoinjury remains unclear.

Previous studies have indicated that the freezing–thawing process results in loss of sperm plasma membrane proteins, changes in sperm membrane composition, and deleterious effects on sperm plasma membrane integrity [13–15]. The plasma membrane disruption leads to leakage of intracellular sperm proteins and the loss of cytoplasmic proteins, membrane-bound proteins, enzymes and other cellular components. Sperm proteins are responsible for sperm function [16]. The loss of sperm proteins may negatively affect fertilization and early embryonic development, and result in male infertility [17]. A promising approach to identify extensive proteins affected by cryopreservation is to investigate the proteomic profile of sperm before and after freezing and thawing. The monitoring of proteinic alteration at the proteomics level in human sperm will benefit our understanding of the mechanism of cryoinjuries resulted from the sperm freezing and thawing process. Proteomics is a powerful tool for discovery of differentially expressed proteins [18, 19]. It has been applied to characterize changes in sperm proteins under various conditions including cryopreservation. Two dimensional gel electrophoresis has been used to detect the proteome of sperm [20]. Recently, the development of liquid chromatography and mass spectrometry has allowed the analysis of proteomes with higher practical throughput strategies. The method used for the quantification of proteins has developed into a combination of isobaric tags for relative and absolute quantification (iTRAQ) and LC–MS/MS [21].

Commercially available egg-yolk free media have been developed and widely used for human sperm freezing [22]. In contrast to conventional egg yolk media, these commercial media contain only chemically defined components, which avoid the potential contamination with animal origin of bacteria, fungi, viruses, and prions, and eliminate the risk of sperm damage by the endotoxins produced by microbes in egg yolk [23]. However, in general, sperm cryopreserved with a chemically defined medium either by using computerized slow-stage freezing or nitrogen vapor fast freezing showed compromised frozen-thawed sperm motility and vitality compared to those cryopreserved with a conventional egg yolk medium [23, 24]. Therefore, cryodamage is still a general and unavoidable phenomenon and an established universal method that can be referred to as an entrenched standard for routine laboratory use is still needed [24]. The progress in proteomics provides a promising method to identify key proteins of sperm that are affected by cryopreservation and explore the mechanism of cryoinjuries which lead to the decline of sperm function and fertilizing ability. A few studies have reported qualitative changes of protein profiles in fish, boar, rat, chicken, ram and human sperm [25–30]. Conventionally, cryostraws (CS) and cryovials (CV) are the most widely used cryopreservation carriers (CPCs) for human sperm cryopreservation accompanied with the use of commercial egg yolk-free media. However, the influence of cryopreservation on the proteomic profile of human sperm preserved with the two CPCs is still unknown.

Therefore, the aims of this study were to (1) compare two different CPCs: cryostraw (CS) and cryovial (CV) on the cryosurvival of human sperm frozen with a chemically defined medium, (2) investigate the proteomic profiles of human sperm cryopreserved with CS and CV, which were evaluated by using iTRAQ techniques, and (3) identify the susceptible proteins that play key roles in sperm function and fertility. Our study will be beneficial for extending the knowledge of cryoinjuries and will provide fundamental information for the development and optimization of human sperm cryopreservation methods.

## Methods

### Ethics statement

The protocol of the present study was approved in advance by the Ethics Review Board of Kunming University of Science and Technology and Kunming Sino-UK Angel Women’s & Children’s Hospital. Informed consent for participation was obtained from all subjects.

### Semen collection and assessment

Semen samples were obtained from a total of 11 healthy male volunteers aged from 26 to 49 years after 7 days of sexual abstinence. All semen samples were collected by masturbation into a sterile container. The semen was allowed to liquefy at 37 °C for at least 30 min, then a routine semen analysis was performed to determine semen volume, sperm concentration, motility, and morphology according to 5th edition of the World Health Organization (WHO) manual (2010). Only the semen samples that met the following criteria: volume ≥ 2.0 mL, sperm concentration ≥ 40 × 10^6^/mL, and progressive motility ≥ 50% were used in this study [31].

### Cryopreservation and thawing of sperm samples

Each completely liquefied semen sample was divided into three aliquots that were referred to as control group (C), cryostraw group (CS) and cryovial group (CV). Semen samples of CS and CV were cryopreserved with Vitrolife’s SpermFreeze Solution (Vitrolife, Sweden) by following the manufacturer’s instructions. Briefly, an equal volume of Vitrolife’s SpermFreeze Solutions were added slowly and dropwise to the semen samples of CS and CV group, and then carefully mixed. The mixtures were equilibrated at room temperature for 10 min and were then sealed into pre-cooled (4 °C) 0.25 ml cryostraws (IMV, L’Aigle, France) (CS group) or 1 ml cryovials (Thermo, USA) (CV group). The cryostraws and cryovials were horizontally and uprightly placed on a Styrofoam board with a 2 cm thickness in a liquid nitrogen bath, respectively. After being held in the liquid nitrogen vapor for 30 min, the semen samples were submerged directly into liquid nitrogen. After a minimum of 7 days of storage in liquid nitrogen, the cryostraw and cryovials were thawed by being plunged directly into a 37 °C water bath for 30 s and 10 min, respectively. Then the post-thaw parameters of motilities were checked.

### Determination of the motility parameters

The motility parameters of fresh sperm (C) and frozen-thawed sperm from CS and CV groups were examined with a computer assisted sperm analyzer (CASA, Sperm Class Analyzer Microptic, Barcelona, Spain) [23]. Each specimen was randomly sampled with at least 600 sperm and the kinetic parameters were evaluated within 5 min with the CASA. The descriptors of sperm motility included percentage of motile sperm (MOT), forward activity rate (FAR), curvilinear velocity (VCL), straight line velocity (VSL), average path velocity (VAP), linearity(LIN), straightness index (STR), vibration index (VIB) and amplitude of lateral head displacement (ALH).

### Extraction of sperm proteins

The semen samples from C, CS, and CV groups were washed 3 times with PBS at 500 g for 10 min. The proteins of sperm collected from the three groups were extracted by the trichloroacetic acid (TCA)/acetone precipitation method. The samples were placed in a mortar and pulverized by addition of liquid nitrogen. Then the powder was placed in a 50 ml centrifuge tube with 10% pre-cooled TCA-acetone solution (containing 0.1% DTT and 1 m M PMSF) at 4 °C overnight. The solution was centrifuged at 12,000*g* for 20 min at 4 °C, and then the supernatant was discarded. The precipitate was re-suspended in the acetone (containing 0.1% DTT and 1 mM PMSF) and was allowed to stand at − 20 °C for 2 h. The solution was centrifuged at 12,000*g* for 20 min at 4 °C again. The precipitate was dried in a freeze-dried vacuum dryer for 30 min. The dry protein powder was stored in a refrigerator at − 80 °C.

### iTRAQ labeling

Each sample contained 100 μg of protein with 5 times the volume of pre-cooled acetone and was at − 20 °C for 1 h. The solution was centrifuged for 20 min at 12,000*g*/min at 4 °C and the supernatant was discarded. The precipitate was then vacuum freeze-dried. After the addition of 50 μL of the Dissolution Buffer in the iTRAQ kit, the samples were mixed thoroughly with a vortex mixer, 4 μL of the Reducing Reagent was added, and the samples were placed at 60 °C for 1 h. Then, 2 μL of Cysteine-Blocking Reagent was added for 10 min at room temperature. The protein solution after each reductive alkylation was pipetted into an ultrafiltration tube (Nanosep MF Centrifugal concentrator). The samples were centrifuged for 40 min at 12,000*g* at 4 °C and the supernatant was discarded. After the addition of 100 μL of the Dissolution Buffer, the samples were centrifuged for 30 min at 12,000*g*. Trypsin (concentration 1 μg/μL) was added to each sample and hydrolyzed at 37 °C for 14 h. After centrifugation (4 °C, 12,000*g*, 40 min), 50 μL of the Dissolution Buffer was added to the ultrafiltration tube and then centrifuged again (4 °C, 12,000*g*, 30 min). The corresponding iTRAQ marker was added to the sample according to the correspondence between the sample and the marker, and the mixture was centrifuged thoroughly and allowed to stand at room temperature for 2 h. Mass spectrometry was performed using the TripleTOF5600 system (SCIEX) combined with the lift-off spray III ion source (SCIEX, USA).

### Selection of the differential proteins

The experimental data were analyzed using Protein Pilot Software v. 5.0 (SCIEX, USA). The standard for confidently identifying a protein is if the protein meets the following; a FDR (false discovery rate) < 1%, Unused > 1.3, and peptides (95%) ≧1. Differential protein screening was based on a trusted protein. The ratio between the samples were at 1.3-fold change (increased) or less than 1/1.3-fold change (decreased) (p < 0.05) and the trend was consistent. Through the comparison of the two experimental groups, we obtained three sets of differential protein spectra, including Cryostraw/Control (CS/C), Cryovial/Control (CV/C), and Cryovial/Cryostraw (CV/CS). UniProtKB database (www.uniprot.org) was used to categorize proteins which were enriched. The official gene symbols of the differentially expressed proteins were used to investigate and categorize the GO annotations. The original GO annotations (cellular components, molecular functions, and biological processes) were downloaded from the NCBI Entrez Gene database and the proteins related to sperm function were selected after further analysis.

### Statistical analysis and bioinformatics analysis

The motility parameters of sperm (MOT, FAR, VCL, VSL, VAP, LIN, STR, VIB and ALH) scored by CASA are presented as mean ± SD. The percentage data for sperm motility underwent arcsine square root transformation before statistical analysis. ANOVA and the Fisher protected least-significant difference test (SPSS 16, SPSS, Chicago, IL) were used to analyze differences among control, CS, and CV groups. A *p* value of less than 0.05 was considered to be statistically significant.

Gene Ontology (GO) enrichment analysis of differentially expressed genes was implemented by GOseq, in which gene length bias was corrected. GO functional analyses provided GO functional classification annotation for DEGs as well as GO functional enrichment analysis for DEGs. GO was generated using the Gene Ontology database (http://www.geneontology.org/). Different genes usually cooperate with each other to exercise their biological functions. Pathway-based analysis helps to further understand these genes biological functions. KEGG is the major public pathway-related database (http://www.genome.jp/kegg/). KOBAS software was used to test the statistical enrichment of differential expression genes in KEGG pathways (*p* value < 0.05).

## Results

### Effect of cryopreservation on the motility parameters of human sperm frozen in cryostraw and cryovial

The motility parameters of human sperm cryopreserved with Vitrolife’s SpermFreeze Solution in cryostraws and cryovials were summarized in Table 1. Compared to fresh control, sperm cryopreserved in either cryostraws or cryovials showed significant decrease in the percentage of MOT, the rate of FAR and the velocity of VCL, VSL and VAP (*p *< 0.05), and significant differences of MOT and FAR were observed between sperm from CS and CV groups (*p *< 0.05). However, the velocity of VCL, VSL and VAP did not differ between the CS and CV groups (*p *> 0.05). In addition, the percentage of LIN, STR and VIB did not differ among the 3 groups (*p *> 0.05).

**Table 1:** Effect of cryopreservation on the motility parameters of human sperm frozen in cryostraw and cryovial

Group	Control (C)	Cryostraw (CS)	Cryovial (CV)	p value		
				C:CS	C:CV	CS:CV
MOT (%)	81.83 ± 5.71^a^	19.10 ± 4.67^b^	27.55 ± 9.60^c^	9.37E−18	3.55E−16	0.01
FAR (%)	63.36 ± 7.65^a^	9.72 ± 3.43^b^	15.48 ± 6.17^c^	1.06E−17	1.85E−16	0.04
VCL (μm/s)	39.26 ± 6.26^a^	27.69 ± 3.98^b^	27.78 ± 2.73^b^	5.11E−06	5.75E−06	0.97
VSL (μm/s)	14.49 ± 2.93^a^	9.31 ± 1.75^b^	9.64 ± 1.87^b^	2.00E−05	4.83E−05	0.75
VAP (μm/s)	23.95 ± 3.43^a^	16.19 ± 2.18^b^	16.29 ± 2.13^b^	5.07E−07	6.31E−07	0.93
LIN (%)	37.13 ± 5.45	33.62 ± 4.22	34.49 ± 4.13	0.10	0.21	0.68
STR (%)	60.33 ± 5.62	57.27 ± 6.63	58.77 ± 4.87	0.24	0.55	0.56
VIB (%)	61.31 ± 3.99	58.78 ± 4.67	58.59 ± 3.83	0.19	0.16	0.92
ALH (μm)	2.09 ± 0.31^a^	1.89 ± 0.28^ab^	1.82 ± 0.14^b^	0.09	0.03	0.54

Different superscripts within a row indicate significant differences (*p* < 0.05)

*MOT* motile sperm; *FAR* forward activity rate; *VCL* curvilinear velocity; *VSL* straight line velocity; *VAP* average path velocity; *LIN* linearity; *STR* straightness index; *VIB* vibration index; *ALH* amplitude of lateral head displacement

### Identification of human sperm proteins

**Fig. 1:**
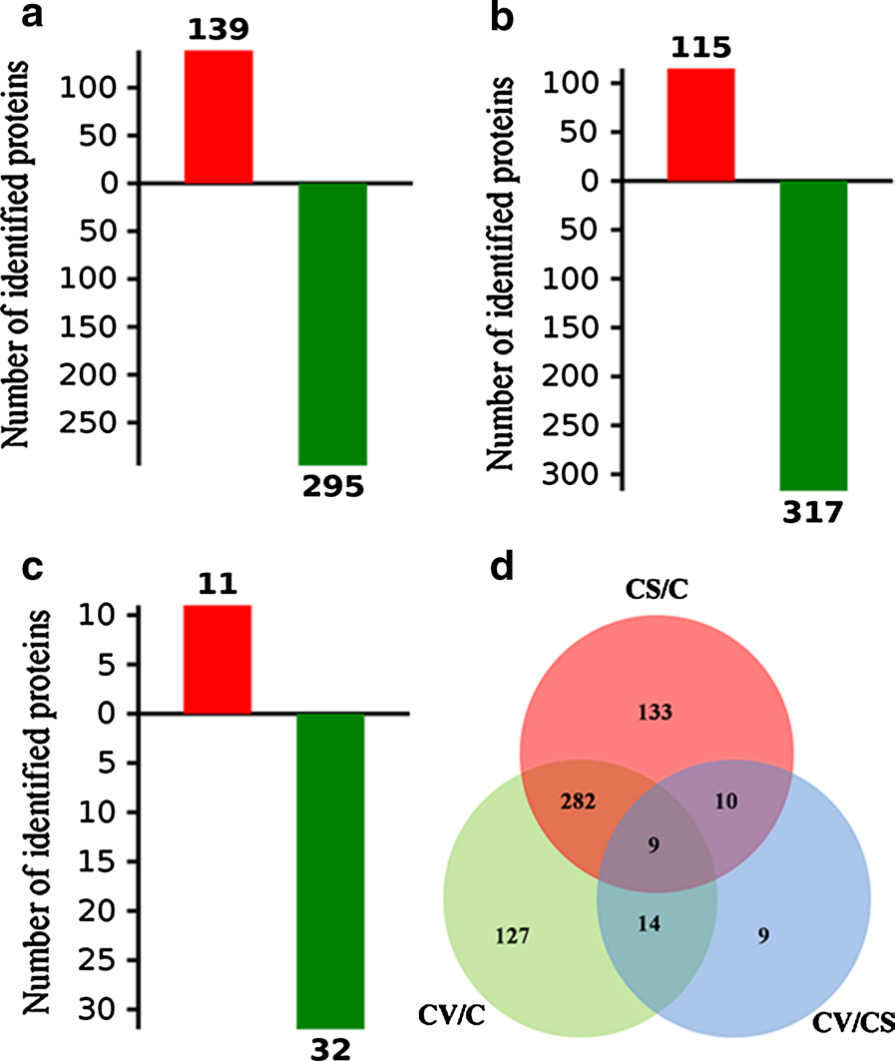
Identification of human sperm differential proteins. Differentially identified human sperm proteins between cryostraw and control (**a**), between cryovial and control (**b**), and between cryovial and cryostraw (**c**) were quantified. The red and green bars represent increased protein and decreased protein, respectively. Venn diagrams show the differences in differentially identified human sperm proteins among the three groups (**d**)

A total of 3294 proteins were identified in human sperm (Additional file 1: Table S1). False Discovery Rates (FDRs) using a reverse concatenated decoy database resulted in estimates of peptide and protein FDR to be smaller than 1%. The differentially identified human sperm proteins among control, CS, and CV are summarized in Fig. 1 and Additional file 2: Table S2. The results showed that after freezing and thawing, the sperm cryopreserved with either cryostraw or cryovial (CS or CV group) presented a large number of changes in sperm proteins compared to those from the non-frozen control group (C group). The results showed that 115 proteins increased and 317 proteins decreased between CS and control (Fig. 1a), 139 proteins increased and 295 proteins decreased between CV and control (Fig. 1b), and 11 proteins increased and 32 proteins decreased between CS and CV (Fig. 1c). According to the Venn diagram analysis of sperm proteomic profile shown in Fig. 1d, a total of 584 identified proteins were differentially distributed among the human sperm from the C, CS and CV groups, and 9 proteins were differentially present in the three groups simultaneously and presented an intersection among the three groups.

### Gene ontology (GO) functional analysis

**Fig. 2:**
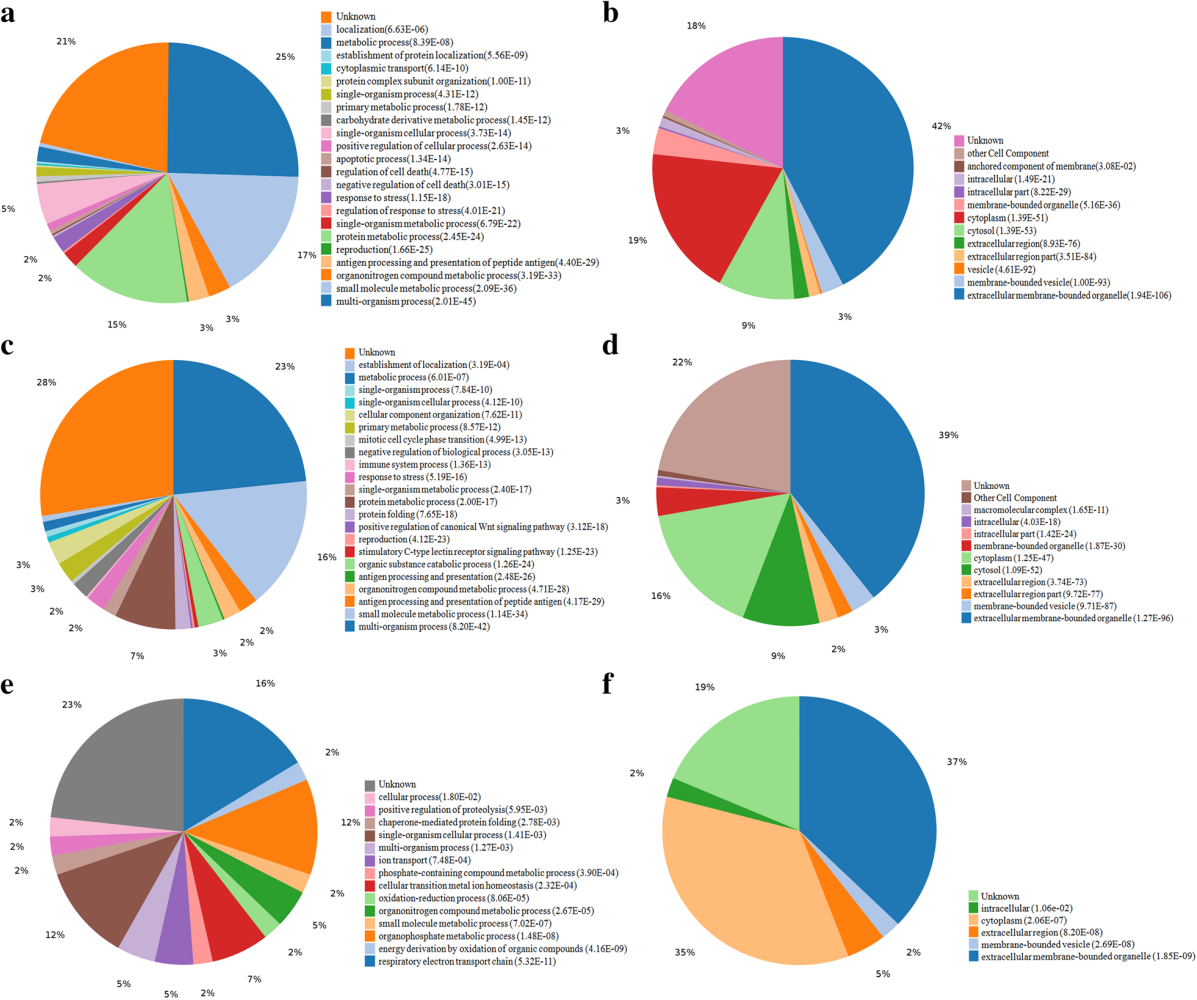
Biological process and cellular localization. Biological process of differential protein, cryostraw/control (**a**) cryovial/control (**c**) and cryovial/cryostraw (**e**). The Differential protein were examined with respect to cellular localization using GO annotation analysis, cryostraw/control (**b**) cryovial/control (**d**) and cryovial/cryostraw (**f**)

The enrichment analysis of Gene Ontology (GO) and the cellular localizations of the identified differential proteins between the C, CS, and CV groups are presented in Fig. 2.

The distribution of biological processes in the ontology of GO terms between C and CS groups were mostly presented in the metabolic processes category, which included small molecule metabolic process (72 proteins, p = 2.09E−36, GO:0044281), organonitrogen compound metabolic process (12 proteins, p = 3.19E−33, GO:1901564), protein metabolic process (64 proteins, p = 2.45E−24, GO:0019538), carbohydrate derivative metabolic process (1 proteins, p = 1.45E−12, GO:1901135), primary metabolic process (3 proteins, p = 1.78E−12, GO:0044238), single-organism metabolic process (9 proteins, p = 6.79E−22, GO:0044710, and other metabolic processes (8 proteins, p = 8.39E−08, GO:0008152). In addition, the distribution of biological terms in the ontology of GO terms were also enriched in proteins that involved in particular cellular processes, including multi-organism process (110 proteins, p = 2.01E−45, GO:0051704), reproduction (1 proteins, p = 1.66E−25, GO:0000003), regulation of response to stress (1 proteins, p = 4.01E−21, GO:0080134), response to stress (9 proteins, p = 1.15E−18, GO:0006950), and cytoplasmic transport (1 proteins, p = 6.14E−10, GO:0016482) (Fig. 2a).

The statistical analysis of functional over-representation showed that the cellular localizations of the identified differential proteins between the C and CS groups are enriched in the extracellular membrane-bounded organelle (184 proteins, p = 1.94E−106, GO:0065010), cytosol (41 proteins, p = 1.39E−53, GO:0005829), and cytoplasm (81 proteins, p = 1.39E−51, GO:0005737) (Fig. 2b).

Similarly, the distribution of biological processes in the ontology of GO terms between C and CV groups were mostly presented in the metabolic processes category, which included small molecule metabolic process (69 proteins, p = 1.14E−34, GO:0044281), organonitrogen compound metabolic process (9 proteins, p = 4.71E−28, GO:1901564), protein metabolic process (32 proteins, p = 2.00E−17, GO:0019538), single-organism metabolic process (7 proteins, p = 2.40E−17, GO:0044710), primary metabolic process (11 proteins, p = 8.57E−12, GO:0044238) and other metabolic processes (5 proteins, p = 6.01E−07, GO:0008152). In addition, the distribution of biological terms in the ontology of GO terms were also enriched in proteins that involved in multi-organism process (101 proteins, p = 8.20E−42, GO:0051704), organic substance catabolic process (13 proteins, p = 1.26E−24, GO:1901575), stimulatory C-type lectin receptor signaling pathway (2 proteins, p = 1.25E−23, GO:0002223), reproduction (1 proteins, p = 4.12E−23, GO:0000003), positive regulation of canonical Wnt signaling pathway (1proteins, p = 3.12E−18, GO:0090263), protein folding (8 proteins, p = 7.65E−18; GO:0006457), response to stress (10 proteins, p = 5.19E−16, GO:0006950), negative regulation of biological process (9 proteins, p = 3.05E−13, GO:0048519) and cellular component organization (12 proteins, p = 7.62E−11, GO:0016043) (Fig. 2c).

The statistical analysis of functional over-representation showed that the cellular localizations of the identified differential proteins between the C and CV groups were enriched in extracellular membrane-bounded organelle (170 proteins, p = 1.27E−96, GO:0065010), cytosol (40 proteins, p = 1.09E−52, GO:0005829) and cytoplasm (71 proteins, p = 1.25E−41; GO:0005737) (Fig. 2d).

The distribution of biological processes in the ontology of GO terms between CS and CV groups included respiratory electron transport chain (7 proteins, p = 5.32E−11, GO:0022904), energy derivation by oxidation of organic compounds (1 proteins, p = 4.16E−09, GO:0015980), organophosphate metabolic process (5 proteins, p = 1.48E−08, GO:0019637), small molecule metabolic process (1 proteins, p = 7.02E−07, GO:0044281), organonitrogen compound metabolic process (2 proteins, p = 2.67E−05, GO:1901564), oxidation–reduction process (1 proteins, p = 8.06E−05, GO:0055114), cellular transition metal ion homeostasis (3 proteins, p = 2.32E−04, GO:0046916), phosphate - containing compound metabolic process (1 proteins, p = 3.90E−04, GO:0006796), ion transport (2 proteins, p = 7.48E−04, GO:0006811), multi-organism process (2 proteins, p = 1.27E−03, GO:0051704), single-organism cellular process (5 proteins, p = 1.41E−03, GO:0044763), chaperone-mediated protein folding (1 proteins, p = 2.78E−03, GO:0061077), positive regulation of proteolysis (1 proteins, p = 5.95E−03, GO:0045862), and cellular process (1 proteins, p = 1.80E−02, GO:0009987) (Fig. 2e).

The statistical analysis of functional over-representation showed that the cellular localizations of the identified differential proteins between the CS and CV groups were enriched in extracellular membrane-bounded organelle (16 proteins, p = 1.85E−09; GO:0065010) and cytoplasm (15 proteins, p = 2.06E−07; GO:0005737) (Fig. 2f).

In order to further analyze the effect of cryopreservation on human sperm function, 106 differential proteins having known identities in sperm functions, according to the UniProtKB database, were identified. The functions of these differentially sperm proteins are listed in Table 2, which are associated with spermatogenesis, iron ion binding, spermatid development, binding of sperm to zona pellucida, sperm capacitation or acrosome reactions, flagellated sperm motility, flagellar microtubules, serine-type endopeptidase inhibitor activity, serine-type peptidase activity and mitochondrial. Specific protein information and the fold of change in different groups were shown in Table 3.

**Table 2:** Biological processes classification of differential identified proteins with known identities of sperm functions

Biological processes	Gene name (Accession)
Iron ion binding	ARSA (P15289), CABYR (O7592), CALR (P27797), CRISP2 (P16562), DPEP3 (Q9H4B8), FKBP1A (P62942), HSP90B1 (P14625), HSPA5 (P11021), NDUFS3 (O75489), PHGDH (O43175), RDH16 (O75452), SEMG1 (P04279), VAT1 (Q99536)
Spermatogenesis	ACE (P12821), ACSBG2 (Q5FVE4), ACTR1A (P61163), CCDC136 (Q96JN2-4), CYLC2 (Q14093), HSF2BP (O75031), HSPA2 (P54652), KRT9 (P35527), MNS1 (Q8NEH6), NUP62 (P37198), OAZ3 (Q9UMX2), PAFAH1B3 (Q15102), PGAM2 (P15259), PRDX4 (Q13162), PSMA1 (P25786), PSMA2 (P25787), PSMA3 (P25788), PSMA4 (P25789), PSMA5 (P28066), PSMB1 (P20618), PSMB4 (P28070), PSMB5 (P28074), PSMB7 (Q99436), RAD23B (P54727), ROPN1B (Q9BZX4), RUVBL1 (Q9Y265), SMRP1 (Q8NCR6), SOD1 (P00441), SPA17 (Q15506), SPANXA1 (Q9NS26), SPATA6 (Q9NWH7), SPEM1 (Q8N4L4), TXNDC2 (Q86VQ3)
Spermatid development	DPY19L2 (Q6NUT2), FSCN3 (Q9NQT6), KLHL10 (Q6JEL2), SPAG6 (O75602-3), SPANXB1 (Q9NS25)
Binding of sperm to zona pellucida	ZPBP1 (Q9BS86), ZPBP2 (Q6X784)
Flagellated sperm motility	CCDC147 (Q5T655), DNALI1 (O14645), DPCD (Q9BVM2), LDHC (P07864), PGK2 (P07205), SMCP (P49901)
Flagellar microtubules	TEKT1 (Q969V4), TEKT2 (Q9UIF3), TEKT3 (Q9BXF9), TEKT4 (Q8WW24), TEKT5 (Q96M29)
Serine-type endopeptidase inhibitor activity	SLPI (P03973), SPINT3 (P49223), WFDC8 (Q8IUA0)
Serine-type peptidase activity	CTSG (P08311), PPP4R1 (Q8TF05), PREP (P48147), PRSS37 (A4D1T9), PRTN3 (U3KPS2)
Sperm capacitation or acrosome reactions	ACR (P10323), ACRBP (Q8NEB7), AKAP3 (O75969), BSPH1 (Q075Z2), C9orf9 (Q96E40), ELSPBP1 (Q96BH3), PRKACA (P17612), SEPT4 (O43236), TCP11 (Q8WWU5)
Mitochondrial	ATP5D (P30049), ATP5H (O75947), ATP5 J (P18859), C21orf33 (P30042), COX4I1 (P13073), COX5B (P10606), COX6B1 (P14854), CYCS (C9JFR7), FSIP2 (Q5CZC0), IMMT (Q16891-4), MPC1L (P0DKB6), MRPS36 (P82909), NDUFA4 (O00483), PHB2 (J3KPX7), VAT1 (Q99536), VDAC3 (Q9Y277)
Other	CCDC108 (Q6ZU64), GNPDA1 (P46926), MRPS36 (P82909), NAMPT (P43490), PATE1 (Q8WXA2), PATE4 (P0C8F1), PGK1 (P00558), PMFBP1 (Q8TBY8-2), SEPHS1 (P49903), STOM (P27105), TSGA10 (Q9BZW7)

**Table 3:** 106 differential proteins associated with sperm function

Protein ID	Protein name	CS/C	CV/C	CV/CS
ACE	Angiotensin-converting enzyme	0.59	0.48	N/A
ACR	Acrosin	0.32	0.39	N/A
ACRBP	Acrosin-binding protein	N/A	0.49	N/A
ACSBG2	Long-chain-fatty-acid–CoA ligase ACSBG2	0.46	0.54	N/A
ACTR1A	Alpha-centractin	0.27	0.18	N/A
AKAP3	A-kinase anchor protein 3	4.07	3.56	N/A
ARSA	Arylsulfatase A	0.48	0.48	N/A
ATP5D	ATP synthase subunit delta, mitochondrial	N/A	1.68	N/A
ATP5H	ATP synthase subunit d, mitochondrial	2.58	N/A	0.82
ATP5 J	ATP synthase-coupling factor 6, mitochondrial	1.94	1.50	N/A
BSPH1	Binder of sperm protein homolog 1	N/A	0.40	0.45
C21orf33	ES1 protein homolog, mitochondrial	N/A	0.45	0.49
C9orf9	Uncharacterized protein C9orf9	2.33	2.32	N/A
CABYR	Calcium-binding tyrosine phosphorylation-regulated protein	0.31	N/A	N/A
CALR	Calreticulin	0.39	0.36	N/A
CCDC108	Coiled-coil domain-containing protein 108	N/A	N/A	N/A
CCDC136	Isoform 4 of Coiled-coil domain-containing protein 136	2.63	2.93	N/A
CCDC147	Coiled-coil domain-containing protein 147	2.35	2.30	N/A
COX4I1	Cytochrome c oxidase subunit 4 isoform 1, mitochondrial	1.99	1.60	N/A
COX5B	Cytochrome c oxidase subunit 5B, mitochondrial	2.60	2.34	N/A
COX6B1	Cytochrome c oxidase subunit 6B1	2.18	N/A	N/A
CRISP2	Cysteine-rich secretory protein 2	0.59	N/A	N/A
CTSG	Cathepsin G	N/A	0.64	N/A
CYCS	Cytochrome c (Fragment)	5.40	3.19	1.25
CYLC2	Cylicin-2	N/A	2.07	N/A
DNALI1	Axonemal dynein light intermediate polypeptide 1	2.99	2.67	N/A
DPCD	Protein DPCD	0.45	0.44	N/A
DPEP3	Dipeptidase 3	0.44	N/A	N/A
DPY19L2	Probable C-mannosyltransferase DPY19L2	6.52	5.44	N/A
ELSPBP1	Epididymal sperm-binding protein 1	4.95	5.02	N/A
FKBP1A	Peptidyl-prolyl cis–trans isomerase FKBP1A	0.47	N/A	N/A
FSCN3	Fascin-3	4.63	3.88	N/A
FSIP2	Fibrous sheath-interacting protein 2	2.17	1.99	N/A
GNPDA1	Glucosamine-6-phosphate isomerase 1	N/A	0.33	N/A
HSF2BP	Heat shock factor 2-binding protein	2.28	N/A	N/A
HSP90B1	Endoplasmin	0.29	0.37	N/A
HSPA2	Heat shock-related 70 kDa protein 2	0.19	0.23	N/A
HSPA5	78 kDa glucose-regulated protein	0.29	0.36	N/A
IMMT	Isoform 4 of Mitochondrial inner membrane protein	2.08	N/A	N/A
KLHL10	Kelch-like protein 10	0.43	0.50	N/A
KRT9	Keratin, type I cytoskeletal 9	7.83	N/A	N/A
LDHC	L-lactate dehydrogenase C chain	0.16	0.19	N/A
MNS1	Meiosis-specific nuclear structural protein 1	N/A	0.48	N/A
MPC1L	Mitochondrial pyruvate carrier 1-like protein	N/A	2.74	N/A
MRPS36	28S ribosomal protein S36, mitochondrial	N/A	0.53	N/A
NAMPT	Nicotinamide phosphoribosyltransferase	0.43	0.70	1.47
NDUFA4	NADH dehydrogenase [ubiquinone] 1 alpha subcomplex subunit 4	2.13	N/A	N/A
NDUFS3	NADH dehydrogenase [ubiquinone] iron-sulfur protein 3, mitochondrial	1.67	N/A	N/A
NUP62	Nuclear pore glycoprotein p62	0.48	0.39	N/A
OAZ3	Ornithine decarboxylase antizyme 3	2.83	3.14	N/A
PAFAH1B3	Platelet-activating factor acetylhydrolase IB subunit gamma	N/A	0.49	N/A
PATE1	Prostate and testis expressed protein 1	2.08	2.11	N/A
PATE4	Prostate and testis expressed protein 4	2.09	1.99	N/A
PGAM2	Phosphoglycerate mutase 2	0.45	0.33	N/A
PGK1	Phosphoglycerate kinase 1	0.40	0.56	N/A
PGK2	Phosphoglycerate kinase 2	0.34	0.38	N/A
PHB2	Prohibitin-2	1.99	N/A	N/A
PHGDH	D-3-phosphoglycerate dehydrogenase	N/A	2.20	N/A
PMFBP1	Isoform 2 of Polyamine-modulated factor 1-binding protein 1	4.38	3.82	N/A
PPP4R1	Serine/threonine-protein phosphatase 4 regulatory subunit 1	0.68	N/A	N/A
PRDX4	Peroxiredoxin-4	N/A	0.41	N/A
PREP	Prolyl endopeptidase	0.57	N/A	N/A
PRKACA	cAMP-dependent protein kinase catalytic subunit alpha	0.43	0.44	N/A
PRSS37	Probable inactive serine protease 37	0.39	N/A	N/A
PRTN3	Myeloblastin	N/A	0.66	N/A
PSMA1	Proteasome subunit alpha type-1	0.58	0.52	N/A
PSMA2	Proteasome subunit alpha type-2	0.32	0.31	N/A
PSMA3	Proteasome subunit alpha type-3	0.65	0.62	N/A
PSMA4	Proteasome subunit alpha type-4	0.40	0.38	N/A
PSMA5	Proteasome subunit alpha type-5	0.45	0.37	N/A
PSMB1	Proteasome subunit beta type-1	0.37	0.35	N/A
PSMB4	Proteasome subunit beta type-4	0.25	0.22	N/A
PSMB5	Proteasome subunit beta type-5	0.41	0.44	N/A
PSMB7	Proteasome subunit beta type-7	0.38	0.44	N/A
RAD23B	UV excision repair protein RAD23 homolog B	0.22	0.31	N/A
RDH16	Retinol dehydrogenase 16	N/A	1.59	N/A
ROPN1B	Ropporin-1B	4.20	N/A	N/A
RUVBL1	RuvB-like 1	0.26	0.26	N/A
SEMG1	Semenogelin-1	N/A	0.55	0.55
SEPHS1	Selenide, water dikinase 1	0.44	0.59	N/A
SEPT4	Septin-4	1.73	N/A	N/A
SLPI	Antileukoproteinase	4.90	N/A	N/A
SMCP	Sperm mitochondrial-associated cysteine-rich protein	N/A	3.08	N/A
SMRP1	Spermatid-specific manchette-related protein 1	1.55	N/A	N/A
SOD1	Superoxide dismutase [Cu–Zn]	0.14	0.17	N/A
SPA17	Sperm surface protein Sp17	2.06	1.88	N/A
SPAG6	Isoform 3 of Sperm-associated antigen 6	2.53	N/A	N/A
SPANXA1	Sperm protein associated with the nucleus on the X chromosome A	N/A	5.43	N/A
SPANXB1	Sperm protein associated with the nucleus on the X chromosome B/F	3.95	4.14	N/A
SPATA6	Spermatogenesis-associated protein 6	3.98	N/A	N/A
SPEM1	Spermatid maturation protein 1	2.69	2.13	N/A
SPINT3	Kunitz-type protease inhibitor 3	N/A	3.25	N/A
STOM	Erythrocyte band 7 integral membrane protein	N/A	0.50	N/A
TCP11	T-complex protein 11 homolog	2.06	N/A	N/A
TEKT1	Tektin-1	2.18	N/A	N/A
TEKT2	Tektin-2	4.35	2.86	1.16
TEKT3	Tektin-3	1.81	N/A	N/A
TEKT4	Tektin-4	2.13	2.05	N/A
TEKT5	Tektin-5	N/A	2.32	N/A
TSGA10	Testis-specific gene 10 protein	10.58	9.59	N/A
TXNDC2	Thioredoxin domain-containing protein 2	0.41	0.37	N/A
VAT1	Synaptic vesicle membrane protein VAT-1 homolog	0.60	0.64	N/A
VDAC3	Voltage-dependent anion-selective channel protein 3	2.41	N/A	N/A
WFDC8	WAP four-disulfide core domain protein 8	3.12	2.39	N/A
ZPBP1	Zona pellucida-binding protein 1	N/A	0.41	N/A
ZPBP2	Zona pellucida-binding protein 2	0.42	0.54	N/A

CS/C, The fold of change between cryostraw (CS) and control (C); CV/C, The fold of change between cryovial (CV) and control (C); CV/CS, The fold of change between cryovial (CV) and cryostraw (CS)

### Pathways analysis and protein interaction

**Fig. 3:**
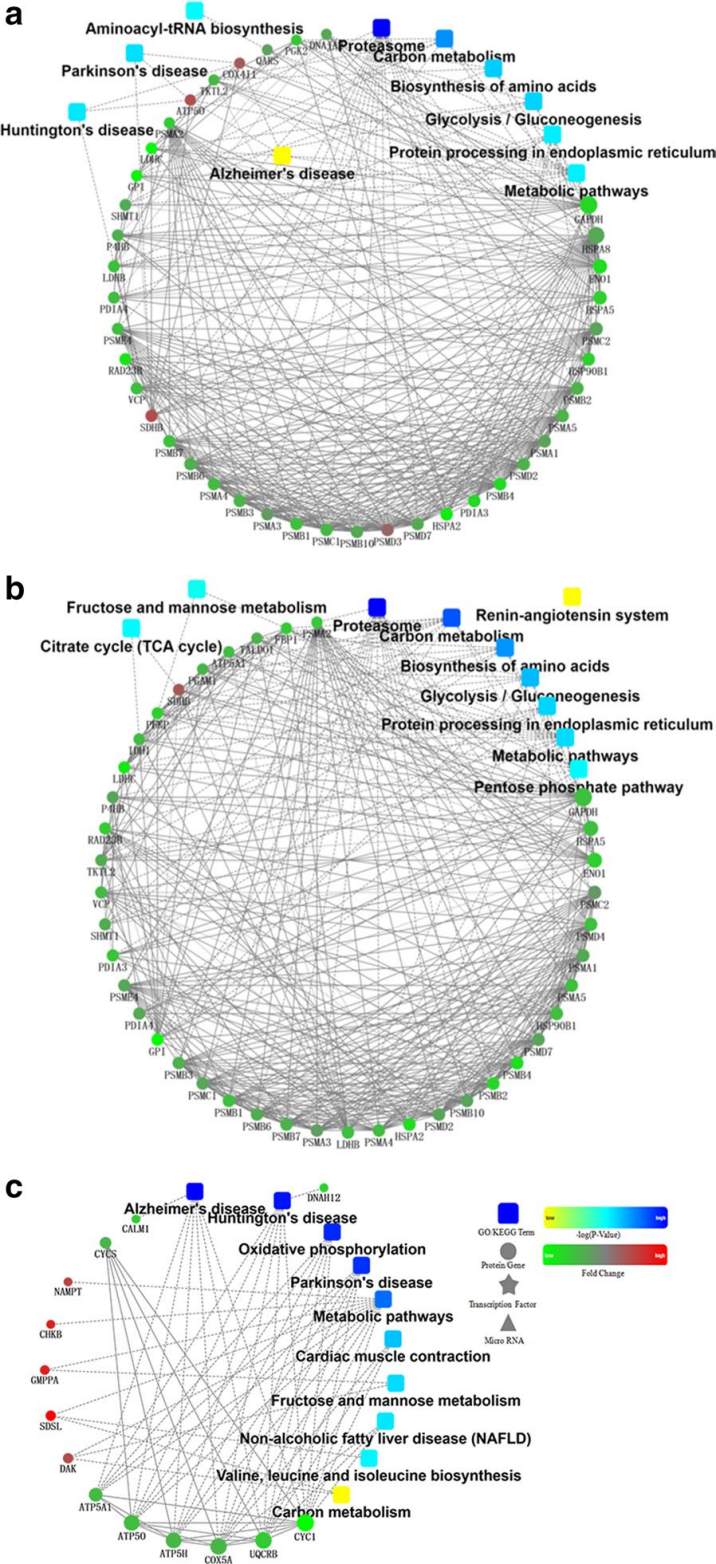
Protein interaction network. Protein interaction network of cryovial/control (**a**), cryovial/control (**b**), cryovial/cryostraw (**c**). 10 pathways were selected statistically enriched in the KEGG pathway (*p* < 0.05). The dots in the figure represent the proteins, the red indicates the increased proteins, and the green represents the decreased proteins. The frame represents the KEGG process. The connection indicates that there is an interaction, the solid line is an interrelated relationship that has been reported, and the dotted line is an unconfirmed interaction

The network of interactions of the differential identified proteins between C and CS groups is shown in Fig. 3a. Ten statistically enriched pathways were selected in the KEGG pathway (*p* < 0.05), which include proteasome (20 proteins), carbon metabolism (20 proteins), biosynthesis of amino acids (13 proteins), glycolysis/gluconeogenesis (12 proteins), Parkinson’s disease (16 proteins), Huntington’s disease (17 proteins), Alzheimer’s disease (14 proteins), protein processing in endoplasmic reticulum (16 proteins), metabolic pathways (57 proteins) and aminoacyl-tRNA biosynthesis (9 proteins).

Similarly, the network of interactions of the differential identified proteins between C and CV groups is shown in Fig. 3b. Ten statistically selected pathways were enriched in the KEGG pathway (*p* < 0.05), which include proteasome (19 proteins), carbon metabolism (22 proteins), biosynthesis of amino acids (15 proteins), glycolysis/gluconeogenesis (12 proteins), protein processing in endoplasmic reticulum (16 proteins), metabolic pathways (53 proteins), pentose phosphate pathway (5 proteins), citrate cycle (TCA cycle) (5 proteins), fructose and mannose metabolism (5 proteins) and renin-angiotensin system (4 proteins).

The network of interactions of the differential identified proteins between CV and CS groups is shown in Fig. 3c. Ten statistically enriched pathways were selected in the KEGG pathway (*p* < 0.05), which includes Alzheimer’s disease (7 proteins), Huntington’s disease (7 proteins), Oxidative phosphorylation (6 proteins), Parkinson’s disease (6 proteins), Metabolic pathways (11 proteins), Cardiac muscle contraction (3 proteins), Fructose and mannose metabolism (2 proteins), Non-alcoholic fatty liver disease (NAFLD) (3 proteins), Valine, leucine, and isoleucine biosynthesis (1 proteins) and Carbon metabolism (2 proteins).

## Discussion

In the present study, we cryopreserved human sperm with an egg yolk-free medium and investigated the effects of cryopreservation using two different CPCs on the cryosurvival of sperm, evaluating motility parameters and the proteome profile of human sperm. After freezing and thawing, the motility parameters of sperm cryopreserved with either cryostraw or cryovial both decreased. However, the sperm cryopreserved in cryovials provided better cryoprotection on motility parameters of MOT and FAR than those preserved in cryostraws. The cryosurvival rate of CS group is consistent with the results reported in previous studies that human sperm cryopreserved in cryostraws showed a relative low cryosurvival rate when using commercial egg yolk free medium [24]. The cooling rate is an important factor that affects the cryosurvival of cells [32]. During freezing, ice nucleates initially in the extracellular spaces and causes an osmotic gradient between the freeze-concentrated extracellular solution and the intracellular solution. Cells are not dehydrated sufficiently when the cooling rate is too fast and intracellular ice formation leads to cryoinjuries in the cytoplasm. Conversely, when the cooling rate is too slow, cells are injured due to solution effects caused by severe dehydration and exposure to toxic concentrations of electrolytes [19]. The optimal freezing rate for sperm cryosurvival should be low enough to avoid intracellular ice formation but fast enough to minimize solution effects [23]. In our study, the sperm were frozen using protocols as suggested by the manufacturer of the sperm freezing medium. The cooling and warming rates of sperm in cryostraws and cryovials might be different during the freezing and thawing processes, which could be the reason that sperm cryopreserved in two CPCs with the same freezing medium showed different sperm cryosurvival rates and motility parameters. Therefore, the optimal cooling rate for human sperm cryopreservation still needs to be determined based on the freezing medium used.

Sperm cryopreservation plays an important role in clinical application of human fertility preservation and infertility treatment. Traditionally, sperm is diluted and equilibrated with commercial egg yolk free freezing medium and is loaded into different CPCs (usually cryostraw or cryovial) which are then frozen in liquid nitrogen vapors. So far, cryodamage is still a general and unavoidable phenomenon. A few studies have reported qualitative changes of protein profiles in fish [25], ram [27], boar [29] and human sperm [30] by proteome analysis, and demonstrated that cryopreservation may result in proteinic alteration of sperm, which is associated with sperm metabolism, membrane permeability, flagella structure and motility, apoptosis, intracellular signaling, capacitation and fertilization commonly [22]. Proteinic alterations of human sperm at proteomic level caused by cryopreservation have been reported previously [22, 33]. Wang and colleagues identified 27 proteins that differed in abundance between fresh and frozen-thawed sperm. However, the proteomic analysis was performed by 2DE and image analysis. Furthermore, the sperm sample was cryopreserved with egg yolk buffered freezing medium containing 10% glycerol, which has been abandoned in clinical applications because egg yolk carries a risk of pathogen introduction into cryopreserved sperm samples. The development of higher throughput strategies for proteome study based on liquid chromatography and mass spectrometry allows detection of the proteinic alterations of sperm with high definition and precision. A previous study found that the abundance of human sperm proteins was altered after being cryopreserved in CV with a protein free medium named CryoSperm and was analyzed via LC–MS/MS. The authors indicated that fewer sperm proteinic changes occurred when semen was thawed in a 23 °C water bath and then maintained after-thawing at 0 °C (60 differential proteins detected) than when it was maintained after-thawing at 23 °C (99 differential proteins detected). In the present study, 139 increased proteins and 295 decreased proteins were detected in sperm cryopreserved with CV, and 115 increased proteins and 317 decreased proteins were detected in sperm cryopreserved with CS. There are several differences in this study as compared to the previous study. Firstly, a different protein-free commercial medium named Vitrolife’s SpermFreeze Solution and freezing protocol were used in our study. Secondly, human semen samples were loaded into two different CPCs (cryostraws or cryovials) for cryopreservation, which are the most commonly used CPCs for human sperm cryopreservation. Lastly, semen samples were thawed in a 37 °C water bath, which is the common thawing method for sperm cryopreservation. These differences may contribute to the variation in proteins identified and quantified. The results also demonstrated that different sperm freezing media, freezing and thawing protocols or CPCs can lead to sperm proteomic profile variations. Cryoinjuries caused by intracellular ice formation lead to sublethal effects on sperm, and influences sperm motility and fertilizing ability [34]. In the present study, the GO analysis revealed that the cellular localizations of the identified differential proteins of human sperm cryopreserved with either CS or CV were enriched in the extracellular membrane-bounded organelles, cytoplasm, and cytosol after cryopreservation. The results agree with previous studies that cryopreservation leads to the leakage of intracellular proteins [35]. The disruption of the sperm membrane integrity resulting in the presence of sperm proteins in extracellular likely reflects damage of the sperm membrane structure [28, 36]. The identification of the biological processes of the identified differential proteins in human sperm cryopreserved with either CS or CV revealed that most of proteins are related to metabolic processes. Proteins of metabolic processes are responsible for the decrease in sperm metabolic activity caused by disturbances in ATP production and ATP regeneration resulting in the decline in sperm motility [37]. Our results indicate that cryoinjuries damage sperm structures, but also decrease sperm metabolism. The latter can lead to a decline in sperm motility, life span, and fertilizing ability. Furthermore, the leakage of mitochondrial proteins from sperm after cryopreservation indicates that disruption of the mitochondrial structure could be responsible for the decrease in energy supply to sperm [30]. Proteasomes can regulate sperm motility through regulation of dynein cAMP-dependent phosphorylation [38]. In addition, the freezing–thawing process reduced the antioxidant capacity of human sperm [39] and lead to disturbances in the ubiquitin–proteasome system, which possibly influenced the motility [40].

Similar to previous studies, increased protein abundance in sperm cryopreserved either with CS or CV was observed [22]. However, the mechanism remains unknown. It was believed that protein phosphorylation is a possible reason for the increased level of some proteins following cryopreservation, which leads to protein degradation, post-translational processing, and alterations in secondary or tertiary structure and/or translocation to other cellular compartments or outside the cell and results in changes in protein abundance [22, 33].

The identified differential proteins of human sperm cryopreserved with either CS or CV were categorized with UniProtKB database including spermatogenesis, spermatid development, flagellated sperm motility, sperm capacitation or acrosome reactions. Differences in the sperm function related proteins were observed between CS and CV groups, which indicate that even if the same freezing medium and the same freezing protocol were used, the effects on sperm proteome can be influenced by the CPCs applied. In addition, we also observed leakages of Ion channel proteins, especially Ca^2+^-binding proteins as shown in the Table 2 including HSPA5 (78 kDa glucose-regulated protein), CABYR (Calcium-binding tyrosine phosphorylation-regulated protein), HSP90B1 (Endoplasmin), CALR (Calreticulin). Calcium ions play a pivotal role in the mechanism controlling human sperm movement [26, 41]. Further studies are necessary to understand the significance of the disturbance to these proteins after cryopreservation with respect to the fertilizing ability of the sperm and embryo development. The network of interactions between the differentially identified proteins was analyzed. Interestingly, the KEGG pathways of Parkinson’s disease, Huntington’s disease, and Alzheimer’s disease were observed in sperm cryopreserved with CS compared to control sperm, however, these results were not observed in sperm cryopreserved with CV. In consideration of the significantly higher value of MOT and FAR in sperm cryopreserved in CV group than CS group, the use of CV for human sperm cryopreservation in clinical application may provide better safety and efficiency.

## Conclusion

In summary, human sperm motility parameters and proteomic profiles are greatly affected by cryopreservation with either type of CPC. GO analysis revealed that most of the differential sperm proteins identified with iTRAQ techniques were enriched in the extracellular membrane-bounded organelles, cytoplasm, and cytosol. In addition, 106 susceptible proteins having known identities in sperm functions were identified. These results provide useful information for insight into the cryoinjury mechanism and will be beneficial for the development and optimization of human sperm cryopreservation strategies.

## Additional files

**Additional file 1: Table S1.** Total of proteins were identified in human sperm.

**Additional file 2: Table S2.** The differentially identified human sperm proteins.

## Abbreviations

CS: cryostraws
CV: cryovials
CPCs: cryopreservation carriers
iTRAQ: isobaric tags for relative and absolute quantification
ATP: adenosine triphosphate
WHO: World Health Organization
MOT: motile sperm
FAR: forward activity rate
VCL: curvilinear velocity
VSL: straight line velocity
VAP: average path velocity
LIN: linearity
STR: straightness index
VIB: vibration index
ALH: amplitude of lateral head displacement
TCA: trichloroacetic acid
FDR: false discovery rate
GO: gene ontology
NAFLD: non-alcoholic fatty liver disease
